# Point Prevalence Survey of Antimicrobial Use in Selected Tertiary Care Hospitals of Pakistan Using WHO Methodology: Results and Inferences

**DOI:** 10.3390/medicina59061102

**Published:** 2023-06-07

**Authors:** Saadia Ambreen, Numrah Safdar, Aamer Ikram, Mirza Zeeshan Iqbal Baig, Ayesha Farooq, Afreenish Amir, Asim Saeed, Farah Sabih, Qadeer Ahsan, Alia Zafar, Palitha Gunarathna Mahipala, Zikria Saleem, Muhammad Salman

**Affiliations:** 1 National Institute of Health, Park Road, Islamabad 45501, Pakistanmaahin1@yahoo.com (A.I.); afreenish.hassan@yahoo.com (A.A.); salman14m@gmail.com (M.S.); 2 World Health Organization, Country Office, Park Road, Islamabad 45501, Pakistanmahipalap@who.int (P.G.M.); 3 The Fleming Fund Country Grant, DAI Office, Beverly Centre, F-6/1, Blue Area, Islamabad 04403, Pakistan; 4 Department of Pharmacy Practice, Faculty of Pharmacy, Bahauddin Zakaria University, Multan 60800, Pakistan; xikria@gmail.com

**Keywords:** point prevalence survey, prescription, anti-microbial utilization, AWaRe classification, antimicrobial resistance

## Abstract

*Background and objectives:* The inappropriate use of antibiotics in hospitals can potentially lead to the development and spread of antibiotic resistance, increased mortality, and high economic burden. The objective of the study was to assess current patterns of antibiotic use in leading hospitals of Pakistan. Moreover, the information collected can support in policy-making and hospital interventions aiming to improve antibiotic prescription and use. *Methodology and materials:* A point prevalence survey was carried out with data abstracted principally from patient medical records from 14 tertiary care hospitals. Data were collected through the standardized online tool KOBO application for smart phones and laptops. For data analysis, SPSS Software was used. The association of risk factors with antimicrobial use was calculated using inferential statistics. *Results:* Among the surveyed patients, the prevalence of antibiotic use was 75% on average in the selected hospitals. The most common classes of antibiotics prescribed were third-generation cephalosporin (38.5%). Furthermore, 59% of the patients were prescribed one while 32% of the patients were prescribed two antibiotics. Whereas the most common indication for antibiotic use was surgical prophylaxis (33%). There is no antimicrobial guideline or policy for 61.9% of antimicrobials in the respected hospitals. *Conclusions:* It was observed in the survey that there is an urgent need to review the excessive use of empiric antimicrobials and surgical prophylaxis. Programs should be initiated to address this issue, which includes developing antibiotic guidelines and formularies especially for empiric use as well as implementing antimicrobial stewardship activities.

## 1. Introduction

Emergence of antibiotic resistance is becoming a major health threat in both hospital and community settings [1,2]. It has resulted in increased morbidity and mortality rates of patients [3]. Moreover, in addition to morbidity and mortality, and increase in healthcare cost and burden on economic growth is also part and parcel of antimicrobial resistance [4,5]. Antimicrobial resistance results into the death of 700,000 people annually across the globe. If decisive actions are not taken, the toll can reach up to 10 million people by 2050 [6], that would culminate into a reduction of 2.5% in GDP worldwide [7]. The unavailability and paucity of data on antimicrobial prescribing are a significant hurdle in formulation and subsequent implementation of antimicrobial stewardship programs globally, especially in low- and middle-income countries (LMICs) [8,9]. Moreover, the relation between antimicrobial resistance and antimicrobial consumption has been well-established, specifically in the case of broad-spectrum antibiotics [10]. Pakistan is among the highest consumers of antibiotics in LMICs and a large increase in consumption was observed among LMICs compared with high-income countries [11].

Taking into consideration the increasing threat of AMR and its global impact, the World Health Organization constituted a Global Action Plan (GAP) in May, 2015 [12]. One of the major goals of GAP is to devise strategies to curb the inappropriate use of antibiotics and ensure its quality use to tackle the threat of AMR [13,14]. In order to fulfill the goals of GAP, particularly objective four that pertains to optimization of the use of antimicrobial agents, point prevalence survey is one strategy to conduct regular surveillance of antibiotic use [9,15]. Point prevalence survey is a practical method to analyze the use of antimicrobials in hospitals and subsequently results can be used in identifying targets for the interventions in quality of antimicrobial use [16]. The availability of data and information is imperative for the policy-making process to curb antimicrobial resistance [17,18].

An estimate states that antimicrobial use, measured in standard units, increased by 35% in the time period of 2000 to 2010 [19]. Moreover, there is growing body of data on the use of antibiotics in hospitals of lower middle-income countries such as Pakistan [20,21,22]. In this regard, a few studies have been published recently in relation to the use of antibiotics in Pakistan, including the irrational use of antibiotics [23]. Nevertheless, no such study using WHO PPS has been published methodology where comparison of patients with or without antibiotics was performed. The results of the study can be used to evaluate quality indicators, to follow-up antimicrobial stewardship and infection control programs, and to support decision-making [24]. The objectives of this PPS was to estimate the prevalence of antimicrobial use in major public and private hospitals of Pakistan. Moreover, the aim was to standardize data collection on hospital-based antimicrobial use and facilitate comparisons across time and between hospitals, regions, and countries. It also provides a standardized tool for hospitals to identify the gaps, and needs for quality improvements, and helps in designing hospital stewardship programs in Pakistan.

## 2. Materials and Methods

### 2.1. Study Design

The study design for this cross-sectional study was WHO PPS methodology, which was applied on selected acute care hospitals across the country.

### 2.2. Study Settings

Fourteen tertiary care hospitals from the public and private sectors designated as GLASS sentinel sites were selected. Hospitals were segregated into three categories according to the number of beds, i.e., <500, 500–1000, and >1000.

### 2.3. Duration

The survey was completed in 3 weeks’ time (October–November 2020).

### 2.4. Sample Size and Sampling Technique

The cases based on hospital records (*n* = 3587) from 14 participating sites were included in the survey.

For the hospitals with <500 beds, samples from all eligible cases were taken. For hospitals with 500 to 800 total inpatient beds, one out of two patients per ward was included. The investigator performed the systematic random sampling and selected the first patient from the list and from this random starting point every second patient was selected until the end of the list was reached. In hospitals with >800 total inpatient beds, one out of three patients per ward were included. After systematic random selection from this random starting point, every third patient was selected until the end of the list was reached.

### 2.5. Survey Tool and Data Collection

A structured questionnaire developed in accordance with the WHO guidelines was adopted, and an investigation team was responsible for gathering all relevant information from eligible cases and submitting it to the hospital coordinator on daily basis as per the methodology. A total of 120 healthcare providers from various departments and units of the hospitals were trained and the survey team included a national coordinator, hospital coordinator, and investigation team. The hospital coordinator was the custodian of the survey data for that particular hospital. He was designated to supervise and monitor the process of data collection, consolidation on daily basis by ensuring the privacy/confidentiality, and sharing with the national coordinator. Data were collected through the standardized online tool KOBO application based on a questionnaire for smart phones. The ‘KOBO Collect’ tool, a web-based application, was used for the first time in Pakistan for the real time data entry, validation, and reporting. Access to this application was provided to all investigation teams, which included physicians, microbiologists, nurses, pharmacists, and infection control practitioners and data managers. Data validation was performed by reviewing records to identify missing information and duplication. Records with incomplete information and duplicate entries were rectified after discussing with duty physicians and nurses, data were also evaluated for consistency and to ensure the reliability using a pre-tested standardized questionnaire. In order to minimize the effect of movement of patients between wards and within the hospital, each ward was completely surveyed within one day.

Antibiotics prescribed to eligible patients in the PPS were enlisted from medical records. The records of all patients meeting the inclusion criteria were enrolled in the PPS irrespective of whether they received antibiotic treatment. Antibiotics were classified according to the Anatomical Therapeutic Chemical Classification (ATC) and AWaRe classification of antibiotics developed by WHO [25,26]. Patients, clinicians, and health facility staff were not interviewed, only the investigation team was engaged to monitor records. In case of retrieval of any missing record, health facility staff was involved.

### 2.6. Inclusion Criteria

∘ Patients in acute tertiary care hospitals registered in GLASS sentinel sites;
∘ Patients hospitalized before or at 08:00 hours on the days of the PPS whether they are receiving antibiotics or not and those patients who have complete medical record;
∘ Data of all patients admitted in acute care settings were included;
∘ Only antibiotics included in list administered by oral, parenteral, rectal, or inhalation routes;
∘ Antibiotic therapy initiated by 08:00 am on survey day.

### 2.7. Exclusion Criteria

∘ Patients from nursing homes, rehabilitation centers, and psychiatric centers are excluded as per WHO PPS methodology;
∘ Patients admitted after 08:00 am on day of survey, all day care patients;
∘ Inpatients from long-term care wards, Emergency departments, day surgery wards, day care wards (e.g., renal dialysis), outpatient clinic, day surgery/day treatment, outpatient dialysis, discharged patients, and waiting for transportation;
∘ Patients receiving ophthalmologic antibiotics.

### 2.8. Analysis Plan

The analysis was mainly focused on use of antimicrobial agents for systemic use in all participating healthcare facilities on the basis of the type of admission specialty, prescription and diagnosis, quality indicators, type of samples, and AWaRe classification. Descriptive analysis was performed by expressing the results in counts, percentages, rates, and proportions. For continuous variables and distribution of data, mean, median, standard deviation (SD), range, and Inter Quartile Range (IQR) were computed. The regression model was applied to determine the association of risk factors with antimicrobial use by computing crude odds ratio at 95% confidence interval (CI) and *p*-value < 0.05. Data were presented in the form of tables, charts, and graphs.

## 3. Results

### 3.1. Prevalence and Demographics

A total of 3587 patients were surveyed, the mean age was 33 years. Out of the total patients, 51% (*n* = 1828) were male and 49% (*n* = 1759) were females. Distribution of admitted patients regarding type of specialty revealed that the majority of patients remained admitted to the surgical ward, 37% (*n* = 1337); followed by medical ward, 36% (*n* = 1276); pediatrics and neonates, 15% (*n* = 521); and 4% each at gynecology, ICU, obstetrics/maternity wards. The median days of stay was 7 days. Among the admitted patients who went through invasive procedures; 80% (*n* = 2871) had a peripheral vascular catheter with or without other invasions, 26% (*n* = 943) had a urinary catheter, 4% (*n* = 155) had a central vascular catheter, and 3% (*n* = 123) had endotracheal intubation (Table 1).

### 3.2. Antibiotic Use and Prescription Rate

Antimicrobials prescription pattern showed that government hospitals had a higher rate of prescriptions, 77% (*n* = 2354) compared with private sector hospitals at 68% (*n* = 347). All hospitals, both private and government sector, had >60% of patients on antimicrobials, except Rawalpindi institute of cardiology had a comparatively lower rate of 46%. Overall, calculated prevalence was 75 per 100 population.

**Figure 1:**
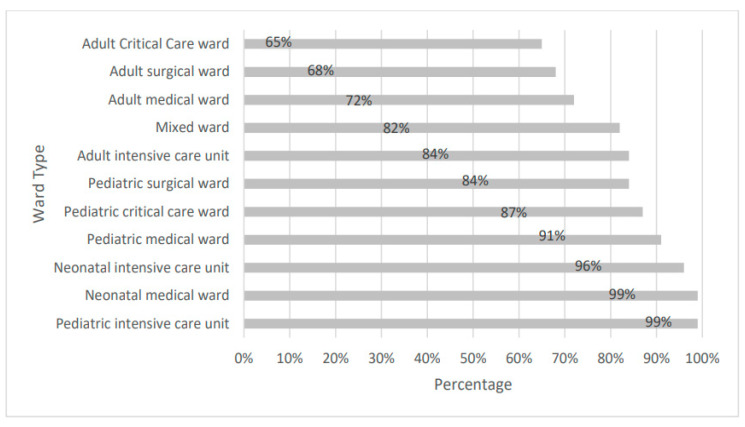
Prevalence of antimicrobial use by the type of ward (*n* = 3587).

Antimicrobial use by the type of ward showed that pediatric intensive care and the neonatal medical ward had higher prevalence (99%) followed by neonatal intensive care (96%) and pediatrics medical ward (91%), the rest were found to have <90% prevalence. The adult critical care ward had the lowest prevalence (65%) (Figure 1).

Antimicrobial prescription pattern by indication shows that third-generation cephalosporin was the most prescribed antibiotic for medical prophylaxis and other indications. Carbapenems were prescribed (14%) mostly for HAI, Penicillin/beta-lactamase inhibitors prescription was very much similar (13–17%) for all indications. Surprisingly the prescription rate of the fluoroquinolone group for all indications was lower (3–8%) compared with other commonly used antibiotics. Anti-mycotic, anti-mycobacterial, anti-viral, anti-malarial, and intestinal anti-infective were rarely prescribed (Table 2).

According to ATC classification, third-generation cephalosporin were prescribed mainly for medical and surgical prophylaxis, unknown infections, community-acquired infections, and healthcare-associated infections. Aminoglycosides were also used in all indications but comparatively less than cephalosporin.

### 3.3. Factors Affecting Compliance

**Figure 2:**
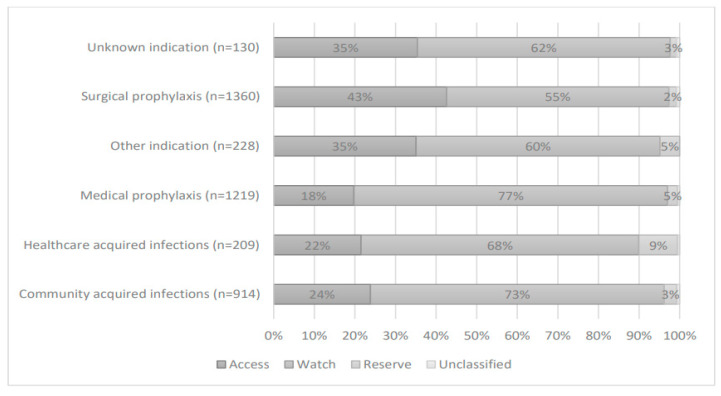
Proportion of prescribed antimicrobials by AWaRe classification (*n* = 4060).

The AWaRe classifies antibiotics into three stewardship groups: access, watch, and reserve to emphasize the importance of their optimal uses and potential for antimicrobial resistance. A survey of antimicrobials use revealed that on average (67%) are on watch, (30%) on access, (3%) reserve, and only a small number of antimicrobials are unclassified (<1%) (Figure 2).

All hospitals had a high rate (>70%) of empirical approach for antimicrobial prescription, whereas the average rate of targeted treatment is merely (7.42%). The highest rate (30%) for the targeted approach was recorded in only one private sector hospital. Among patients with the antimicrobial indication of infection (both HAIs and CAIs), 94% of treatment was empirical and 6% of treatment was targeted (TS-1). To find the association of a risk factor with antimicrobial use, the crude odds ratio (OR) was computed that may have a confounding effect, OR can be over- or underestimated due to confounders. The detailed values are mentioned in the table (Table 3 and Table S1).

## 4. Discussion

Point prevalence studies help the health department understand the current situation about the prescribing trends of antimicrobial agents [9,27]. Moreover, such scientific evidence guides to adopt a rational and appropriate choice of antimicrobials for both therapeutic and prophylaxis treatment in healthcare facilities at all levels [28]. Strategies are designed and planned in such a focused and targeted manner to reduce the incidence and future prevention. This is especially important for countries such as Pakistan where the burden of infectious diseases is constantly on the rise.

This point prevalence survey was conducted in Pakistan to investigate antimicrobial usage among the hospitalized patients in major tertiary care hospitals in both the public and private sector. The results of the PPS survey in 14 hospitals from different provinces/regions of Pakistan revealed the overall calculated prevalence of antimicrobial use in hospitals inpatients is 75%, the result of this survey is comparable with African countries in the global PPS (50%) [29]. The observed prevalence of antibiotic use in African countries’ PPS studies revealed prevalent antibiotic use in Kenya (67.7%), Botswana (70.6%), Ghana (60.5%), and Jordan (75.6%). In contrast, a lower prevalence was observed in the following: 43% in Australia, 56% in China, 32.9% in Europe, 38% in Canada, and 34.4% from the reported data collected across 53 countries [8].

The highest antimicrobial use was found in the neonate intensive care unit in this study. The prevalence of AMU was 99% in the pediatric intensive care unit in the public sector hospital, and 100% in private hospitals. Likewise, as reported in other PPS studies conducted among the adult population in Nigeria, Pakistan, and Brazil, high prevalence of antimicrobial use in ICU compared with other hospital inpatient wards was due to the critical health status of patients in the intensive unit, serious infections, and co-existing medical conditions [9,14]. Moreover, high frequency of hospital-acquired infections in low- and middle-income countries leads to prolonged and higher antimicrobial usage in the intensive care unit of the hospital [30,31].

The third-generation cephalosporin and metronidazole were the most commonly prescribed antimicrobials, these antimicrobials were prescribed for community-acquired infections, healthcare-associated infections, and surgical prophylaxis. One of the reasons is that these agents are easily available and less expensive [32]. Broad-spectrum antimicrobials use was also reported in hospitals of sub-Saharan Africa [33]. Use of the carbapenem group and vancomycin (glycopeptides) is very limited and restricted because these are the last-line treatment options for severe invasive infections and much more costly [34]. One reason for the frequent use of third-generation cephalosporin in the pediatric ward is due to the fact that children admitted with serious infections such as pneumonia, gastroenteritis, and meningitis, etc., tend to receive doses of broad-spectrum antimicrobials without confirmation of AST [35].

Antimicrobials were prescribed frequently for the treatment of different infections empirically without microbiologic or other examinations of samples from patients that would have guided for targeted treatment as only a small number of patient samples were taken for culture and drug susceptibility analyses before initiation of antimicrobials. The reason behind the low culture and sensitivity analysis is the cost issue borne by patients and hospitals. In addition to this, poor capacity and unavailability of laboratory facilities also hinders targeted treatment regime. This is an important finding as targeted therapies are essential to optimize therapy with antimicrobials compared with empirical therapies [36,37].

Medical and surgical prophylaxis for >1 day was a common practice in all surveyed hospitals, which is not in line with the current guidelines which promotes that antimicrobials prophylaxis should only be provided for at least 1–2 h before surgery, medical prophylaxis has the same criteria of limited use of prophylaxis. Different studies have also supported limited use of surgical prophylaxis to achieve the desired level of antimicrobials during surgery [38,39,40].

Compliance to the institutional antimicrobials guidelines was low in this study, 24% (*n* = 1015), the utmost reason can be lack of awareness and unavailability of national guidelines, low capacitated laboratories from where isolates can be timely identified and tested for appropriate drug regimen, and training of antimicrobials prescribers, i.e., physicians to sensitize on rational use of antimicrobials. Moreover, the unavailability of guidelines and local prescription practices can be other reasons for non-compliance with guidelines [14,41,42,43]. Most importantly, there is no such monitoring mechanism that can identify and highlight the deviation from standard protocols for corrective action. Findings will be communicated to all participating hospitals, post-survey sensitization and awareness will enhance the compliance rate.

A statistically significant association was found between following variables with more chances of receiving antimicrobials; neonates (OR 10.9), pediatrics (<18 years) (OR 2.3), having NHSN surgery (OR 5.4), and admitted in pediatrics and neonates specialty (OR 7.39). In a univariate analysis we found that going through certain invasive procedures such as having a central vascular catheter (OR 5.6), peripheral vascular catheter (OR 7.2), endotracheal tube (OR 6.64), or a urinary catheter (OR 3.03) and receiving antibiotics has significant association. In many studies, most of the invasive procedures have a strong association with antimicrobial use, which may be due to long stay, severity of infection, and because such patients are more prone to hospital-acquired infections. Increased rate of AMU among children is due to the unpredictable conditions due to immature immune systems. Resultantly, in some cases infectious diseases may progress to a fatal outcome [44]. Therefore, to avoid such an outcome most of the clinicians opt for an empirical approach instead of going for AST to guide specific treatment. Severity of illness in pediatrics and neonates requires broad-spectrum antimicrobials [45]. The urinary tract infections and frequent complications due to catheterizations are common nosocomial infections [46].

Only a small number of hospitals had patients on antimicrobials classified in the Reserve category of the WHO AWaRe classification; however, the large number of antimicrobials prescribed belonged to the watch group and access group. Higher use of antimicrobials in the watch group can be challenging [27]. The resistance rate in Pakistan is high, which may explain the prevalent use of the watch group. Resistance rates of greater than 50% were observed in *Klebsiella pneumoniae* to third-generation cephalosporins and it was 30–50% in *Escherichia coli* to third-generation cephalosporins, respectively, as reported in the studies [47]. Hospital-specific formularies with the AWaRe classification were taken into consideration to address this challenge. Increasing the use of access agents and limiting or restricting the use of reserve agents can be performed by obtaining authorization from the pharmacist prior to consumption of antimicrobials [48]. Information regarding AWaRe classification must be clearly communicated to all prescribers and posted on a wall. Increasing age was associated with slightly decreased adjusted odds of being on an antibiotic [49]. According to international antibiotic policy, 90% of antibiotic prescriptions should be in accordance with the standard guidelines [50], which basically highlights the need to improve this factor. The average rate of compliance to the guidelines in Pakistan is 30.5% approximately, which is comparatively low. In comparison, published work reports antibiotic compliance to guidelines in Malaysia as (50.4%) [50], and Australia as (67.3%) [51]. This deviation from indicates the underutilization of the protocols established in the hospitals, as well as the lack of standard protocols and guidelines tailored according to the local requirements and local custom antibiograms.

Nevertheless, there are a few limitations to this study. This survey was designed in a way to access records and was restricted to a brief interview with the physicians and patients to explore some more in-depth information and core issues. However, there was limited availability of patient data in hospital records, due to which detailed insight was not captured. The data were not compared with the prescribed antibiotics against proven or suspected microbiology results. Moreover, this PPS was conducted in the short time duration which reflects one point time only. The survey was conducted for only three weeks’ time, so we might have lost some important information of patients admitted on other days. Another important limitation was the selection bias because selection of hospitals was based on convenience rather than randomization. Moreover, GLASS sentinel sites were selected, which have microbiology diagnostic support which affects prescription practices compared with other sites; hence, the findings cannot be generalized. The majority of the sites were from capital cities of provinces and not even a single site from Baluchistan province was enrolled in the survey. Hospitals in sub-urban areas, rural settlements, and other parts of the province were missing. Therefore, we cannot generalize the results of this survey.

## 5. Conclusions

The AMR challenge is quite huge in Pakistan, and there is a dire need to implement a pragmatic stewardship program in all public and private sector tertiary care hospitals for continuous training and awareness of physicians on rational use of antimicrobials. A standard PPS program should be introduced in routine antimicrobial stewardship programs (ASPs) hospital-wide, where possible. AMS programs should be designed to prevent the deviation of therapy from standard protocols, particularly in the prescription of broad-spectrum antibiotics, non-oral antibiotics, and antibiotics used for surgical prophylaxis. One of the key components of AMS programs is improving laboratory capacity permitting targeted therapy and prudent use of antibiotics. Moreover, training of physicians on WHO AWaRe classification, development, and dissemination of national guidelines can be one of the strategies to improve rational use of antimicrobials. To preserve the future effectiveness of antibiotics and reduce patient harm due to AMR, it is imperative to collaborate with the provincial health department for provision of resources and implementation of legislation. Until the establishment of a comprehensive and sustainable surveillance system across the country, such PPS should be repeatedly conducted on a larger scale, with a true representation of population and incorporating the epidemiological aspect to understand the core issues triggering escalated AMR. The scientific evidence will help to design targeted and focused interventions to counter this emerging threat.

## Figures and Tables

**Table 1:** General characteristics of surveyed patients by admission specialty.

Characteristics	Total	
	No.	%
No. of surveyed patients	**3587**	-
Mean age of surveyed patients ± SD	33.4 ± 22.5	
Gender		
Male	1828	51%
Female	1759	49%
Admission specialty		
Gynecology	151	4%
ICU	152	4%
Medical	1276	36%
Obstetrics/maternity	150	4%
Pediatrics and Neonates	521	15%
Surgery	1337	37%
Length to stay until day of PPS, days median (IQR)	7 (0–399)	
Surgery since admission	735	21%
Use of invasive devices		
Central vascular catheter	155	4%
Peripheral vascular catheter	2871	80%
Endotracheal tube	123	3%
Urinary catheter	943	26%

**Table 2:** Frequency of different antimicrobials regimen by use (*n* = 4151).

Antimicrobials	Antibiotic Use *N* (%)
Ceftriaxone	1206 (44.0%)
Metronidazole	484 (18.0%)
Amoxicillin/Clavulanic acid	302 (11.0%)
Piperacillin/Tazobactam	297 (10.9%)
Amikacin	257 (9.50%)
Meropenem	200 (7.4%)
Cefoperazone + Sulbactum	183 (6.77%)
Vancomycin	164 (6.0%)
Cefotaxime	151 (5.59%)
Moxifloxacin	132 (4.88%)

**Table 3:** Association of risk factors with antimicrobial use (*n* = 3587).

Factors	Received AM (*n* = 2701)	Did Not Receive AM (*n* = 886)	Total	OR	95% CI	*p* Value
	No. (%)	No. (%)	No.			
Age						
Adults (≥18 yrs.)	1850 (71)	760 (29)	2610	Ref		
Pediatrics (<18 yrs.)	666 (85)	119 (15)	751	2.3	1.86–2.84	0.000
Neonates (≤1 month)	185 (96)	7(4)	192	10.9	5.1–23.2	0.000
Gender						
Male	1381 (76)	447 (24)	1828	Ref		
Female	1320 (75)	439 (25)	1759	0.97	0.84–1.13	0.726
Surgery (*n* = 3580)						
No Surgery	2035 (72)	810 (28)	2845	Ref		
Non-NHS (National Healthcare Safety Network) Surgery	174 (83)	37 (17)	211	1.87	1.3–2.69	0.001
NHSN Surgery	488 (93)	36 (7)	524	5.4	3.8–7.6	0.000
Median Length of stay	4	3		1.012	1.003–1.022	0.012
Admission Specialty						
Medical	909 (71)	367 (29)	1276	Ref		
Gynecology	108 (72)	43 (28)	151	1.01	0.7–1.47	0.942
ICU	126 (83)	26 (17)	152	1.96	1.26–3.04	0.003
OBS and maternity	120 (80)	30 (20)	150	1.61	1.06–2.45	0.025
Pediatrics and Neonates	494 (95)	27 (5)	521	7.39	4.92–11.09	0.000
Surgery	944 (71)	393 (29)	1337	0.97	0.82–1.15	0.722
Invasive procedures						
Central vascular catheter (*n* = 155)	146 (94)	9 (6)	155	5.6	2.83–10.96	0.000
Peripheral vascular catheter (*n* = 2871)	2403 (84)	468 (16)	2871	7.2	6.02–8.61	0.000
Endotracheal tube (*n* = 123)	117 (95)	6 (5)	123	6.64	2.9–15.14	0.000
Urinary catheter (*n* = 943)	830 (88)	113 (12)	943	3.03	2.45–3.76	0.000
Hospital size						
Less than 500	456 (74)	160 (26)	616	Ref		
From 500 to 1000 beds	738 (82)	167 (18)	905	1.55	1.21–1.98	0.000
More than 1000 beds	1507 (73)	559 (27)	2066	0.95	0.77–1.16	0.594
Hospital Type						
Government	2354 (77)	719 (23)	3073	Ref		
Private	347 (68)	167 (32)	514	0.63	0.52–0.78	0.000
Referred from other hospital (*n* = 191)	173 (91)	18 (9)	191	3.3	2.02–5.4	0.000

## Data Availability

Data are available upon reasonable request from the corresponding author.

## Supplementary Materials

The following supporting information can be downloaded at: https://www.mdpi.com/article/10.3390/medicina59061102/s1, Table S1: Comparison of Targeted vs empirical treatment in Surgical Prophylaxis, HAI and CAIs.
