# Supplementary material for: Point Prevalence Survey of Antimicrobial Use in Selected Tertiary Care Hospitals of Pakistan Using WHO Methodology: Results and Inferences
Source: Medicina (Kaunas). 2023 Jun 7;59(6):1102. doi: 10.3390/medicina59061102 (PMC10303015; doi:10.3390/medicina59061102)
Supplement: Supplementary file 1 [file medicina-59-01102-s001.zip › medicina-2376349-supplementary.pdf]

Description	No. of Antimicrobials (%)
**No. of surgical patients**	**735**
**No. of surgical patients prescribed AM**	662 (90)
**No. of surgical patients prescribed AM for SP (n=662)**	573 (87)
**No. of prescribed antimicrobials for surgical prophylaxis**	**1365***
**Pre-surgical prophylaxis**	971 (71.13)
**Post-Surgical prophylaxis (n=1164, 201=missing)**	1049 (90.1)
**Type of treatment (For CAI & HAI)**	**1172**
**Empirical**	1098 (94)
**Targeted**	74 (6)

Table S1: Comparison of Targeted vs empirical treatment in Surgical Prophylaxis, HAI and CAIs
